# Metformin Inhibits Cardiac Fibroblast Differentiation by Promoting Fatty Acid β-Oxidation: Implications for Age-Associated Cardiac Fibrosis

**DOI:** 10.3390/cells15151408

**Published:** 2026-08-04

**Authors:** Hridya Chempon, Sunita Kumari, Srinivasa Reddy Bonam, Srigiridhar Kotamraju

**Affiliations:** 1 Department of Applied Biology, CSIR-Indian Institute of Chemical Technology, Hyderabad 500007, India; hridyacdas@gmail.com (H.C.); sunitaraisr28@gmail.com (S.K.); bsrpharmacy90@gmail.com (S.R.B.); 2 Academy of Scientific and Innovative Research, Ghaziabad 201002, India

**Keywords:** metformin, CPT1, FAO, cardiac fibrosis, senescence

## Abstract

**Highlights:**

**What are the main findings?**

- Metformin enhances FAO to suppress cardiac fibroblast activation.
- Metformin preserves mitochondrial bioenergetics in cardiac fibrosis.

**What are the implications of the main findings?**

- Metformin reduces cardiac fibrosis by enhancing CPT1-dependent FAO.

**Abstract:**

Cardiac fibrosis is a hallmark of pathological cardiac remodeling, characterized by fibroblast activation, excessive extracellular matrix deposition, and myocardial hypertrophy, ultimately leading to cardiac dysfunction. Aging exacerbates these processes through metabolic stress and impaired mitochondrial bioenergetics. Here, we investigated the anti-fibrotic effects of metformin and the role of fatty acid β-oxidation (FAO) in regulating cardiac fibroblast differentiation. Metformin significantly attenuated transforming growth factor-β (TGF-β)-induced cardiac fibroblast activation and the associated senescence-like phenotype. These effects were accompanied by enhanced FAO and increased mitochondrial oxygen consumption rate (OCR), indicating improved mitochondrial function. Importantly, inhibition of carnitine palmitoyltransferase-1 (CPT1) with etomoxir largely abolished the beneficial effects of metformin on mitochondrial respiration, fibroblast activation, and cellular senescence, demonstrating a critical role for FAO. Mechanistically, metformin increased CPT1 activity and acetyl-CoA levels while reducing malonyl-CoA accumulation, thereby promoting mitochondrial fatty acid utilization. These findings were corroborated in aged Apoe^−/−^ mice, where metformin reduced the expression of cardiac fibroblast differentiation markers and enhanced FAO-associated markers. Collectively, our findings demonstrate that metformin suppresses cardiac fibroblast differentiation and senescence by preserving mitochondrial bioenergetics through FAO-dependent mechanisms, revealing a metabolic basis for its anti-fibrotic actions and supporting its therapeutic potential in age-related cardiovascular disease.

## 1. Introduction

Cardiovascular diseases (CVDs) remain the leading cause of mortality worldwide, and aging is a major risk factor for their development and progression. Age-associated cardiac remodeling is characterized by myocardial hypertrophy, increased ventricular stiffness, impaired cardiac function, and excessive extracellular matrix (ECM) deposition [1]. Among these changes, myocardial fibrosis is a key pathological feature that contributes to cardiac dysfunction, arrhythmias, and heart failure. Cardiac fibrosis is primarily driven by the activation and differentiation of resident cardiac fibroblasts into myofibroblasts, a process largely mediated by transforming growth factor-β (TGF-β) [2,3].

Accumulating evidence suggests that aging promotes fibrotic remodeling through mitochondrial dysfunction, oxidative stress, chronic inflammation, and cellular senescence [4]. Given the high energy demand of the heart, maintenance of mitochondrial function is essential for cardiac homeostasis. The adult myocardium derives the majority of its ATP from mitochondrial fatty acid β-oxidation (FAO), and impairment of this pathway is increasingly recognized as a hallmark of cardiac aging [5,6]. Reduced FAO contributes to metabolic stress, diminished ATP production, and mitochondrial dysfunction, all of which may influence fibroblast activation and pathological remodeling. However, the role of FAO in regulating cardiac fibroblast differentiation during aging still remains elusive.

Mitochondrial FAO is tightly regulated by carnitine palmitoyltransferase-1 (CPT1), the rate-limiting enzyme responsible for transporting long-chain fatty acids into mitochondria for β-oxidation [7]. Aging has been associated with reduced expression and activity of CPT1 and other FAO-related enzymes, resulting in impaired mitochondrial bioenergetics [8]. Emerging studies suggest that metabolic reprogramming is a critical determinant of fibroblast fate and function [9], raising the possibility that restoration of FAO may represent a novel strategy to limit cardiac fibrosis.

AMP-activated protein kinase (AMPK) serves as a central regulator of cellular energy homeostasis and promotes FAO through inhibition of acetyl-CoA carboxylase (ACC), thereby relieving malonyl-CoA-mediated suppression of CPT1. Notably, AMPK activity declines with aging, contributing to metabolic dysfunction and reduced mitochondrial efficiency [10]. Metformin, a widely prescribed antidiabetic drug, activates AMPK and has gained attention for its cardioprotective and geroprotective properties. In our previous studies, metformin was shown to modulate AMPK-mediated H3K79 methylation, which in turn improves mitochondrial function and attenuation of endothelial cell senescence [11,12]. Additionally, in a recent study, we showed that metformin administration delays endothelial cell senescence by potentiating FAO [13]. In addition to improving metabolic homeostasis, metformin was shown to attenuate fibrosis, oxidative stress, and cellular senescence in several pathological settings [14]. However, whether the anti-fibrotic effects of metformin are mediated through restoration of FAO-dependent mitochondrial bioenergetics remains unclear. Addressing this knowledge gap is important because current understanding of metformin-mediated cardioprotection has largely focused on modulation of canonical signaling pathways, whereas the contribution of metabolic substrate utilization, particularly FAO, to fibroblast regulation remains poorly characterized. Determining whether FAO restoration represents a functional mediator of metformin’s anti-fibrotic effects may provide new insights into metabolic approaches for preventing age-related cardiac remodeling.

In the present study, we investigated the role of FAO in metformin-mediated regulation of cardiac fibroblast activation and senescence. Using TGF-β-stimulated cardiac fibroblasts and aged Apoe^−/−^ mice, a model with features of hypercholesteremia and age-associated cardiovascular dysfunction, we examined the involvement of the AMPK–ACC–CPT1 signaling axis in fibroblast differentiation. Our findings demonstrate that metformin suppresses fibroblast activation and senescence by enhancing CPT1-dependent FAO and preserving mitochondrial bioenergetics. These results identify FAO as a critical regulator of cardiac fibroblast fate, and reveal a previously underappreciated metabolic mechanism underlying the anti-fibrotic actions of metformin in age-related cardiac fibrosis.

## 2. Materials and Methods

### 2.1. Isolation of Mouse Cardiac Fibroblast Cells

Male 6–8-week-old C57BL/6 mice were sacrificed by cervical dislocation, and hearts were collected after perfusion with PBS. The hearts were transferred to a 60 mm dish, atria were removed, and ventricles were cut in to small pieces and collected. A volume of 2 mL collagenase II solution (Cat. #C6885, Sigma, St. Louis, MO, USA) was added per heart, and the tissue was triturated with a 1 mL pipette tip with a wide opening. The supernatant was collected into a 50 mL centrifuge tube and kept for incubation at 37 °C with continuous shaking for 20 min. Collagenase was inactivated by adding FBS (Cat. #12800017, GIBCO, Paisley, UK) (2 mL per heart), and the cell suspension was transferred to a 15 mL centrifuge tube and centrifuged for 1 min. The pellet was resuspended in DMEM (Cat. #D7777, Sigma, St. Louis, MO, USA) with 10% FBS. Collagenase II solution was added to the remaining tissue and incubated again at 37 °C for 20 min. The above steps were repeated until the tissue was completely dissolved. All of the collected supernatants were clubbed and centrifuged, and the pellet was homogenously suspended in DMEM containing 10% FBS and seeded in Petri dishes. Media was changed after 2 h. After expansion, primary cardiac fibroblasts were passaged, and all experiments were performed using second-passage (P2) cells. Experiments were conducted according to the guidelines formulated for the care and use of animals in scientific research (ICMR, New Delhi, India) at a CPCSEA (Committee for the Purpose of Control and Supervision of Experiments on Animals) registered animal facility. The IAEC approved the experimental protocol at CSIR-IICT (IICT/IAEC/040/2023).

### 2.2. Real Time-qPCR

Total RNA was extracted using TRIzol reagent (Cat. #T9424, Sigma, St. Louis, MO, USA). The purified RNA was subsequently reverse-transcribed into cDNA using a commercially available cDNA synthesis kit (Cat. #K1622, Thermo Fisher Scientific, Waltham, MA, USA). Quantitative real-time PCR (qPCR) was performed using SYBR Green PCR Master Mix (Cat. #4309155, Thermo Fisher Scientific, Waltham, MA, USA) along with gene-specific primers. Relative gene expression levels were determined using the ΔΔCt method, with 18S rRNA serving as internal reference genes for normalization. Details of the gene-specific primer sequences are listed in Table 1.

### 2.3. Western Blot Analysis

Cells were lysed using RIPA buffer (Cat. #R0278, Sigma, St. Louis, MO, USA) supplemented with protease inhibitor cocktail (Cat. #P8340, Sigma, St. Louis, MO, USA), phosphatase inhibitor cocktail-2 (Cat. #P5726, Sigma, St. Louis, MO, USA), and phosphatase inhibitor cocktail-3 (Cat. #P0044, Sigma, St. Louis, MO, USA). Equal amounts of protein were separated on 8–15% SDS-PAGE gels and subsequently transferred onto nitrocellulose membranes. The membranes were incubated overnight at 4 °C with specific primary antibodies, followed by incubation with the corresponding secondary antibodies for 1 h at room temperature. Protein bands were visualized using Luminal/Enhancer solution (Millipore, Hercules, CA, USA) and detected with the ChemiDoc XRS+ imaging system (Bio-Rad, Hercules, CA, USA). The primary antibodies used for Western blot analysis were as follows: CPT1 (Cat. #97361S, Cell Signaling Technology, Danvers, MA, USA), AMPK (Cat. #2795S, Cell Signaling Technology, Danvers, MA, USA), p-AMPK (Cat. #2535S, Cell Signaling Technology, Danvers, MA, USA), ACC (Cat. #3676S, Cell Signaling Technology, Danvers, MA, USA), p-ACC (Cat. #3661S, Cell Signaling Technology, Danvers, MA, USA), α-SMA (Cat. #ab5694, Abcam, Cambridge CB2 0AX, UK), p21 (Cat. #2947S, Cell Signaling Technology, Danvers, MA, USA), p27 (Cat. #SAB4500068, Sigma, St. Louis, MO, USA), and TGF-β (Cat. #3711S, Cell Signaling Technology, Danvers, MA, USA).

### 2.4. Carnitine Palmitoyltranferase-1 (CPT1) Activity

Carnitine palmitoyltransferase-1 (CPT1) enzymatic activity was determined as previously described [15,16]. Briefly, active mitochondrial fractions were isolated using a commercially available mitochondrial isolation kit (Cat. #MITOISO2, Sigma, St. Louis, MO, USA) according to the manufacturer’s instructions. Equal amounts of mitochondrial protein were incubated in a reaction mixture containing 116 mM Tris-HCl (pH 8.0), 0.09% Triton X-100, 1.1 mM EDTA (Cat. #E5134, Sigma, St. Louis, MO, USA), 0.12 mM DTNB (Cat. #D8130, Sigma, St. Louis, MO, USA), 0.035 mM palmitoyl-CoA (Cat. #P9716, Sigma, St. Louis, MO, USA), and either 1 mM L-carnitine (Cat. #8.40092, Sigma, St. Louis, MO, USA) or 11 mM D-carnitine (Cat. No. #HY-W012550, MedChemExpress, Monmouth Junction, NJ, USA). The reaction was initiated by adding mitochondrial fraction, and the formation of CoA-SH was continuously monitored for 4 min at 412 nm in a multimode reader.

### 2.5. Measurement of Acetyl-CoA and Malonyl-CoA Levels

Acetyl-CoA and malonyl-CoA levels were measured using commercially available kits from Sigma (Cat. #MAK039, Sigma, St. Louis, MO, USA) and Krishgen biosystems (Cat. # KBH5628, Krishgen biosystems, Mumbai, India), respectively, as per the manufacturers’ instructions.

### 2.6. Analysis of Mitochondrial Respiration

Mitochondrial function was assessed using an extracellular flux analyzer (XF24; Seahorse Biosciences/Agilent, Santa Clara, CA, USA). Adult mouse cardiac fibroblast cells were seeded at a density of 40,000 cells per well in DMEM. After 16 h, the culture medium was replaced with Seahorse assay medium (Cat. #103575-100, Agilent, CA, USA) supplemented with 10 mM glucose (Cat. #G8270, Sigma, St. Louis, MO, USA), 1 mM pyruvate (Cat. #S8636, Sigma, St. Louis, MO, USA), and 200 mM glutamine (Cat. #G7513, Sigma, St. Louis, MO, USA), followed by incubation for 1 h at 37 °C in a CO_2_-free incubator for equilibration. Etomoxir (40 μM), oligomycin (1 μM), FCCP (carbonyl cyanide-p-trifluoromethoxyphenylhydrazone; 1 μM), and Antimycin A+ Rotenone (1 μM each) (Cat. #103270-100, Agilent, Santa Clara, CA, USA) were loaded into Ports A, B, C, and D, respectively. In a separate experiment, adult mouse cardiac fibroblast cells were plated at the same density in DMEM medium and allowed to attach for 8 h. The medium was subsequently replaced with substrate-limited growth medium containing 0.5 mM glucose (Cat. #G8270, Sigma, St. Louis, MO, USA), 1 mM glutamine (Cat. #G7513, Sigma, St. Louis, MO, USA), 1% FBS (Cat. #12800017, GIBCO, Paisley, UK), and 0.5 mM carnitine (Cat. #103693-100, Agilent, Santa Clara, CA, USA), and the cells were incubated in a CO_2_ incubator for 16 h. Prior to the assay, the medium was exchanged with substrate-limited assay medium supplemented with 2 mM glucose (Cat. #G8270, Sigma, St. Louis, MO, USA) and 0.5 mM L-carnitine (Cat. #103693-100, Agilent, Santa Clara, CA, USA), followed by incubation for 1 h in a non-CO_2_ incubator. Immediately before initiating the assay, 85 μL of 1 × palmitate-BSA (170 μM) (Cat. #103693-100, Agilent, Santa Clara, CA, USA) was added to each well. The oxygen consumption rate (OCR) was determined from the slope of concentration change versus time. Maximal respiration was calculated by subtracting the rotenone and antimycin A-inhibited OCR from the FCCP-stimulated OCR, while ATP production was estimated from the reduction in basal OCR following oligomycin treatment. Coupling efficiency (%) was calculated by dividing ATP production/basal respiration × 100.

### 2.7. Animal Experiments

Animal studies were performed using 12-month-old male Apoe^−/−^ mice according to the guidelines formulated for the care and use of animals in scientific research (ICMR, India) at a CPCSEA (Committee for the Purpose of Control and Supervision of Experiments on Animals) registered animal facility. The IAEC approved the experimental protocols at CSIR-IICT (IICT/IAEC/024/2022). All animals were housed in a specific pathogen-free (SPF) animal facility under controlled environmental conditions (23 ± 1 °C, 50–60% relative humidity) with a 12 h light/12 h dark cycle and had ad libitum access to standard chow diet and water. A group of 6-month-old male Apoe^−/−^ mice was included as the young control. The 12-month-old Apoe^−/−^ mice were randomly assigned to two groups (*n* = 10 per group). To evaluate the adequacy of the sample size used, we performed a post hoc sensitivity analysis using G*Power 3.1. One group was administered with metformin (50 mg/kg body weight/day) prepared in 0.5% methylcellulose containing Tween-20, while the other served as the age-matched vehicle control. Both young control group (6-month-old Apoe^−/−^ mice) and age-matched control group (12-month-old Apoe^−/−^ mice) received only 0.5% methylcellulose and Tween-20. Treatments were given once daily by oral gavage for 12 weeks. Throughout the study, all mice were maintained on a standard chow diet containing 11.4% fat. Daily food intake was monitored, and body weight was measured weekly.

### 2.8. Lipid Staining by Nile Red Assay

Cardiac fibroblast cells or heart tissue sections were stained with Nile Red (1 µg/mL; Cat. #72485, Sigma, St. Louis, MO, USA) for 15 min to visualize lipid content. Following staining, sections were washed three times with PBS to remove excess dye. Fluorescence images were captured using a fluorescence microscope equipped with a rhodamine filter.

#### 2.8.1. Measurement of Cardiac TGF-β Levels by ELISA

TGF-β levels in the cardiac tissue homogenates were quantified using a commercially available ELISA kit (Cat. #KLM0285, Krishgen biosystems, Mumbai, India) according to the manufacturer’s instructions.

#### 2.8.2. Masson’s Trichrome Staining

Masson’s trichrome staining was performed using a commercial staining kit (Cat. #AB150686, Abcam, Cambridge CB2 0AX, UK) according to the manufacturer’s instructions. Images were captured using a bright-field microscope.

#### 2.8.3. Sirius Red/Fast Green Collagen Staining

Collagen staining was performed using a Sirius Red/Fast Green collagen Staining Kit (Cat. #9046, Chondrex Inc., Redmond, WA, USA) following the manufacturer’s instructions. Images were captured using a bright-field microscope.

#### 2.8.4. Hydroxyproline Assay

Collagen content was determined using a Hydroxyproline Assay Kit (Cat. #E-BC-K062-S, Elabscience, Houston, TX, USA) according to the manufacturer’s instructions. Briefly, heart tissue was hydrolyzed with 6 mol/L HCl to release free hydroxyproline. The hydrolysates were reacted with the assay reagents to produce a colored complex, and the absorbance was measured at 558 nm to assess the collagen content.

### 2.9. PKH 26 Staining

Cardiac fibroblasts were washed with PBS and stained with 2 µM PKH26 (Cat. #HY-D1451, MedChemExpress, Monmouth Junction, NJ, USA) according to the manufacturer’s instructions. After staining, cells were washed thoroughly with PBS to remove excess dye and maintained in complete culture medium. Images were acquired using a laser scanning confocal microscope.

### 2.10. Senescence-Associated β-Galactosidase (SA-β-Gal) Staining

Cellular senescence was assessed using a Senescence β-Galactosidase Staining Kit (Cat. #9860, Cell Signaling Technology, Danvers, MA, USA) according to the manufacturer’s instructions. Briefly, cells were washed with PBS, fixed with a fixative solution for 10–15 min at room temperature, and washed twice with PBS. Cells were then incubated with freshly prepared β-galactosidase staining solution at 37 °C in a dry incubator (without CO_2_) overnight. Senescent cells exhibiting blue coloration were visualized under a bright-field microscope and images were captured.

### 2.11. Statistical Analysis

All of the experiments were performed in biological triplicates, and the results are represented as mean ± SD. Data normality and the equality of variances were examined by Shapiro–Wilk test and Brown–Forsythe test, respectively. Data were analyzed by Welch’s ANOVA followed by Dunnett’s T3 post hoc test for unequal variances, and one-way or two-way ANOVA followed by Tukey’s multiple-comparison test for equal variances using GraphPad Prism 10.

## 3. Results

### 3.1. Metformin Enhances CPT1 Activity and Fatty Acid β-Oxidation in Adult Mouse Cardiac Fibroblast Cells

**Figure 1:**
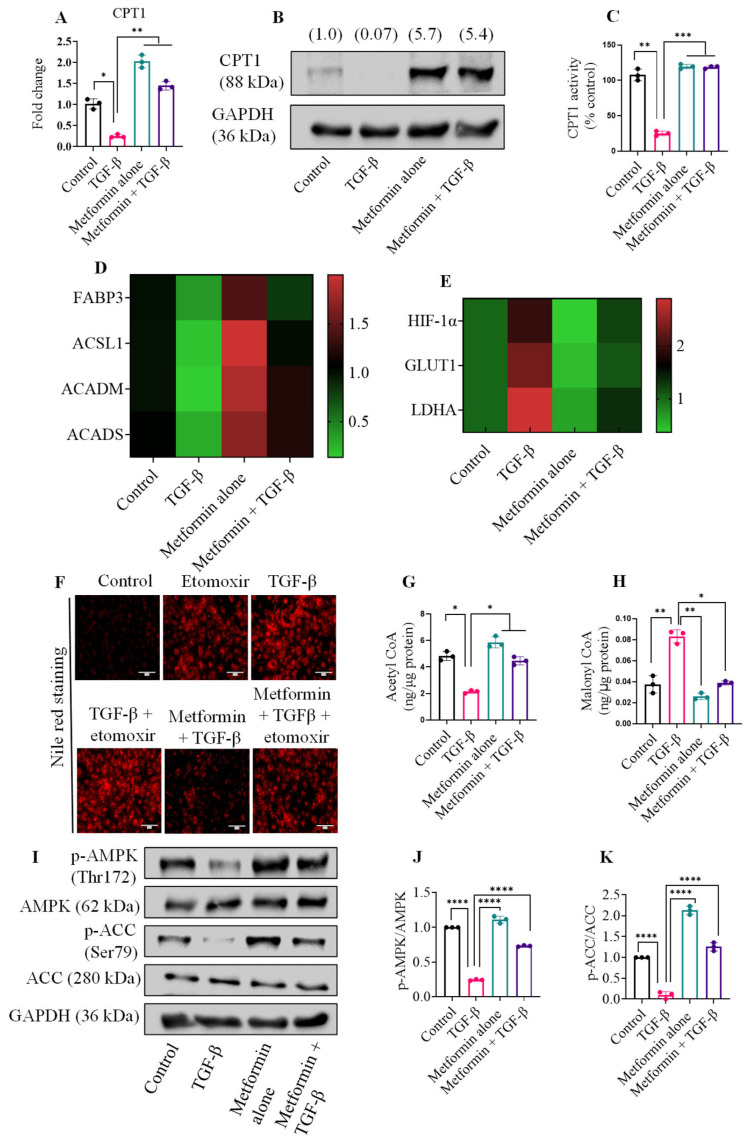
Metformin promotes fatty acid β-oxidation in adult mouse cardiac fibroblasts. (**A**–**C**) Adult mouse cardiac fibroblast cells were pretreated with or without metformin (2 mM) for 12 h followed by TGF-β (10 ng/mL) for 48 h, and CPT1 transcript levels (**A**), protein levels (**B**), and enzyme activity (**C**) were measured. (**D**) Same as *A*, except that transcript levels of *FABP3*, *ACSL1*, *ACADM*, and *ACADS* were measured by RT-qPCR analysis. (**E**) Same as *A*, except that transcript levels of *HIF-1α*, *GLUT1*, and *LDHA* were measured by RT-qPCR analysis. (**F**) Adult mouse cardiac fibroblast cells were pretreated with metformin (2 mM) followed by treatment with TGF-β (10 ng/mL) in the presence or absence of etomoxir (40 µM) for 48 h; lipid accumulation was measured by Nile Red staining, and images were taken in a fluorescence microscope equipped with a rhodamine filter, scale bar = 175 µm. (**G**,**H**) Same as *A*, except that acetyl-CoA levels (**G**) and malonyl-CoA levels (**H**) were measured in cell lysates. (**I**) Same as *A*, except that the indicated protein levels were measured by immunoblot analysis. (**J**,**K**) Quantification of (**I**). GraphPad Prism 10 was used for statistical analysis. Data are presented as mean ± SD from at least three independent experiments. *, *p* ≤ 0.05; **, *p* ≤ 0.005; ***, *p* ≤ 0.0005; ****, *p* ≤ 0.0001. Welch’s ANOVA followed by Dunnett’s T3 post hoc test for unequal variances and one-way ANOVA followed by Tukey’s multiple-comparison test for equal variances, depending on variance homogeneity.

Fatty acid β-oxidation (FAO) is the primary pathway for fatty acid catabolism, generating acetyl-CoA for cellular energy production. Activated cardiac fibroblasts have been reported to exhibit impaired oxidative metabolism and reduced FAO [17]. Given our previous finding that chronic metformin treatment enhances FAO in endothelial cells [13], in the current study, we investigated whether metformin could counteract TGF-β-induced alterations in FAO in primary adult mouse cardiac fibroblasts. For this, cells were treated with TGF-β (10 ng/mL) in the presence or absence of metformin (2 mM) for 48 h. Cells were treated with metformin 12 h prior to the addition of TGF-β. It is to be noted that, intriguingly, while TGF-β alone caused a marked reduction in CPT1 levels, addition of metformin to these cells significantly enhanced the transcript and protein levels of CPT1 (Figure 1A,B). These results were further corroborated by measuring the CPT1 activity, a rate limiting enzyme of FAO (Figure 1C). On a similar note, metformin treatment also led to an increase in the transcript levels of other FAO pathway-related genes (Figure 1D). On the contrary, metformin treatment significantly attenuated TGF-β-induced upregulation of the glycolysis-associated genes (Figure 1E). Collectively, these findings suggest that metformin reverses TGF-β-induced metabolic alterations by restoring FAO while suppressing glycolysis-associated genes expression. Based on the above observations, we next performed Nile Red staining and observed a significant reduction in the intracellular lipid content with metformin + TGF-β treatment conditions compared to TGF-β alone treatment conditions. In contrast, etomoxir (an irreversible inhibitor of CPT1) reversed metformin-mediated reduction in the lipid levels (Figure 1F). This observation suggests that the reduction in the intracellular lipid content in metformin-treated cells is perhaps due to the increased consumption of fatty acids by FAO. Moreover, this result coincides with the increased levels of acetyl-CoA, a key metabolic intermediate and end product of fatty acid β-oxidation, along with a reduction in the levels of malonyl-CoA, a well-established endogenous inhibitor of CPT1-dependent mitochondrial fatty acid uptake, in metformin-treated cells (Figure 1G,H).

**Figure 2:**
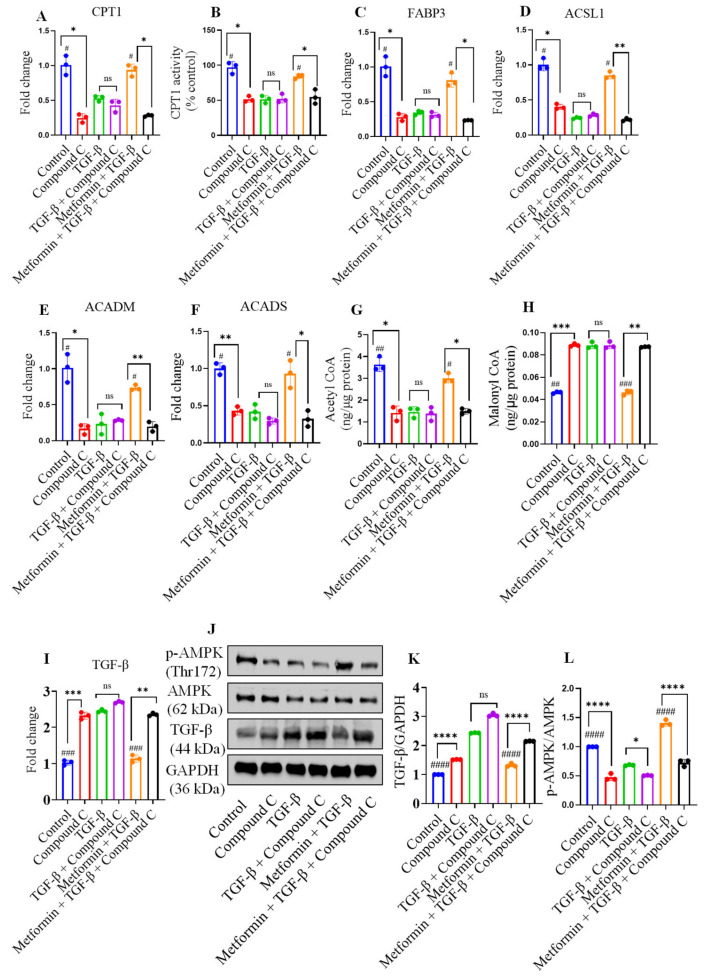
Inhibition of AMPK activation abolishes metformin-mediated enhancement of fatty acid β-oxidation in cardiac fibroblasts. (**A**,**B**) Adult mouse cardiac fibroblasts were treated with Compound C (10 μM) in the presence or absence of metformin (2 mM) for 12 h, followed by TGF-β (10 ng/mL) stimulation for 48 h, and CPT1 transcript levels (**A**) and enzyme activity (**B**) were measured. (**C**–**F**) Same as *A,* except that transcript levels of *FABP3*, *ACSL1*, *ACADM*, and *ACADS* were measured by RT-qPCR analysis. (**G**,**H**) Same as *A,* except that acetyl-CoA levels (**G**) and malonyl-CoA levels (**H**) were measured in cell lysates. (**I**) Same as *A,* except that transcript levels of *TGF-β* (**I**) and the indicated protein levels (**J**) were assessed by RT-qPCR and Western blot analysis, respectively. (**K**,**L**) Quantification of (**J**). GraphPad Prism 10 was used for statistical analysis. Data are presented as mean ± SD from at least three independent experiments. ns is non-significant, *, *p* ≤ 0.05; **, *p* ≤ 0.005; ***, *p* ≤ 0.0005; ****, *p* ≤ 0.0001. #, *p* ≤ 0.05; ##, *p* ≤ 0.005; ###, *p* ≤ 0.0005; ####, *p* ≤ 0.0001 significantly different from TGF-β. Welch’s ANOVA followed by Dunnett’s T3 post hoc test for unequal variances and one-way ANOVA followed by Tukey’s multiple-comparison test for equal variances, depending on variance homogeneity.

Furthermore, to investigate the upstream signaling cascade by which metformin regulates fatty acid β-oxidation, we assessed the activation of AMPK and ACC by immunoblotting. Acetyl-CoA carboxylase (ACC) catalyzes the conversion of acetyl-CoA to malonyl-CoA, a key metabolite that inhibits CPT1-mediated mitochondrial fatty acid uptake. However, phosphorylation of ACC by activated AMPK inhibits its enzymatic activity, thereby reducing malonyl-CoA levels and relieving CPT1 inhibition. Metformin significantly enhanced the levels of phospho-AMPK (Thr-172) with a concomitant increase in phospho-ACC (Ser-79; an inhibitory phosphorylation) compared to TGF-β alone treated cardiac fibroblast cells (Figure 1I–K). Taken together, these findings indicate that metformin promotes FAO in adult mouse cardiac fibroblasts. To determine whether AMPK activation is required for the above observed effects of metformin, cardiac fibroblasts were treated with Compound C, a pharmacological inhibitor of AMPK. Inhibition of AMPK noticeably attenuated the metformin-induced increase in CPT1 transcript levels and its enzyme activity (Figure 2A,B). Consistent with this observation, the transcript levels of other FAO-associated genes were also significantly reduced in Compound C-treated cells (Figure 2C–F). In addition, inhibition of AMPK activation decreased intracellular acetyl-CoA levels while increasing malonyl-CoA levels (Figure 2G,H), indicating suppression of fatty acid β-oxidation. These results demonstrate that activation of the AMPK–ACC axis is essential for metformin-mediated restoration of FAO in TGF-β-stimulated cardiac fibroblasts. Moreover, under these conditions, metformin treatment significantly reduced TGF-β expression, whereas pharmacological inhibition of AMPK with Compound C abolished this effect, as demonstrated by both RT-qPCR and Western blot analyses (Figure 2I–L). Notably, treatment with Compound C alone significantly increased TGF-β levels relative to the untreated control, suggesting that basal AMPK activity negatively regulates TGF-β expression. Together, these results support the conclusion that AMPK activation contributes to the suppressive effect of metformin on TGF-β levels in cardiac fibroblast cells.

### 3.2. Metformin Potentiates Mitochondrial Respiration by Enhancing FAO in Adult Mouse Cardiac Fibroblast Cells: Effect of CPT1 Inhibition

**Figure 3:**
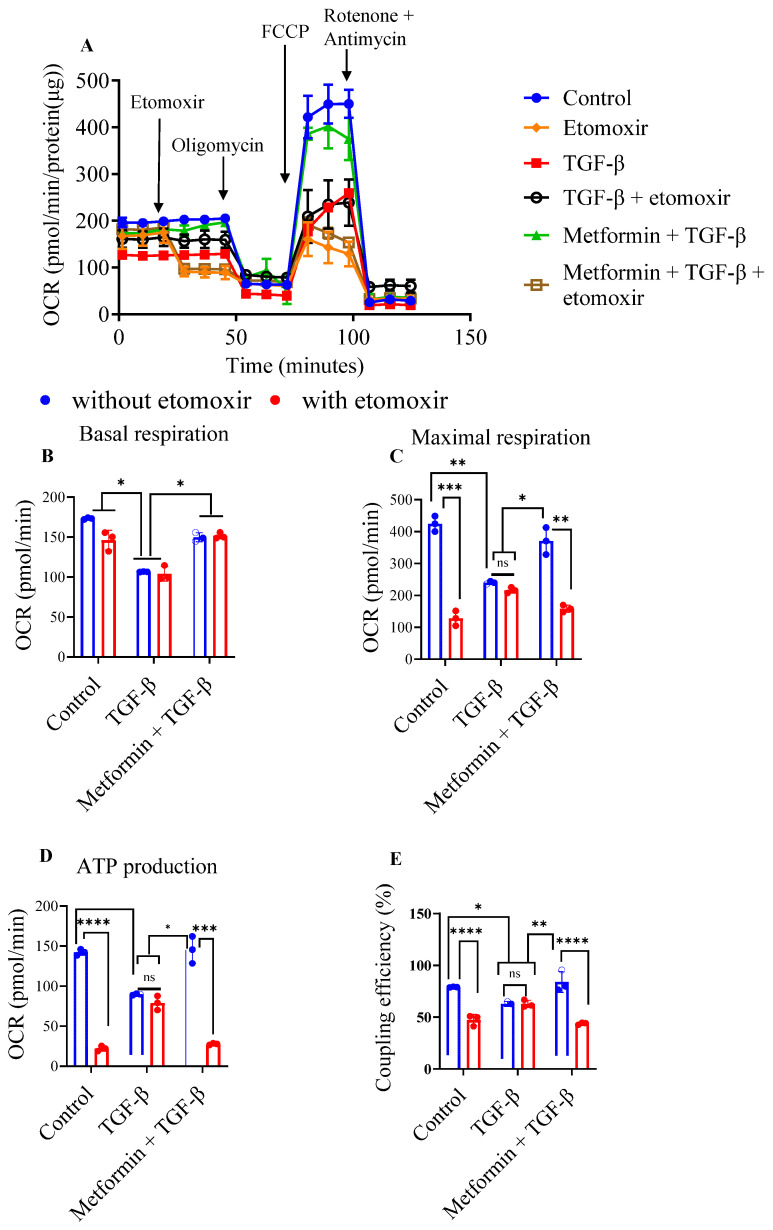
Etomoxir reverses metformin-mediated increase in mitochondrial oxygen consumption rate (OCR) in adult mouse cardiac fibroblast cells. (**A**) Adult mouse cardiac fibroblast cells were pretreated with or without metformin (2 mM) for 12 h followed by TGF-β (10 ng/mL) for 48 h, and the oxygen consumption rates (OCR) were measured by sequentially adding (indicated by arrows) Etomoxir (40 µM), Oligomycin (1 µM), FCCP (1 µM), Rotenone (1 µM), and Antimycin (1 µM) using a Seahorse XF 24 Extracellular Flux Analyzer. (**B**–**E**) Same as A, except that basal respiration (**B**), maximal respiration (**C**), ATP production (**D**), and coupling efficiency (**E**) were measured from the data obtained in A. GraphPad prism 10 was used to perform the statistics. The data shown in the graphs represent the mean ± SD of at least three different experiments. ns is non-significant, *, *p* ≤ 0.05; **, *p* ≤ 0.005; ***, *p* ≤ 0.0005; ****, *p* ≤ 0.0001 by two-way ANOVA followed by Tukey’s multiple-comparison test.

Since metformin enhanced CPT1 activity and FAO-related transcript levels like *FABP3*, *ACSL1*, *ACADM*, and *ACADS*, we next assessed its functional relevance by measuring mitochondrial oxygen consumption rate (OCR) using the Seahorse extracellular flux analyzer. Cardiac fibroblast cells were treated with TGF-β alone or in combination with metformin, in the presence or absence of Etomoxir (CPT1 inhibitor). Cells treated with TGF-β exhibited a significant reduction in the basal respiration, maximal respiration, ATP production, and coupling efficiency compared to untreated control cells. Notably, pretreatment with metformin significantly improved the aforementioned parameters (Figure 3A–E). Additionally, in accordance with the results presented in Figure 1, metformin-mediated increases in OCR, ATP production, maximal respiration, and coupling efficiency were markedly attenuated in the presence of etomoxir (Figure 3A–E).

**Figure 4:**
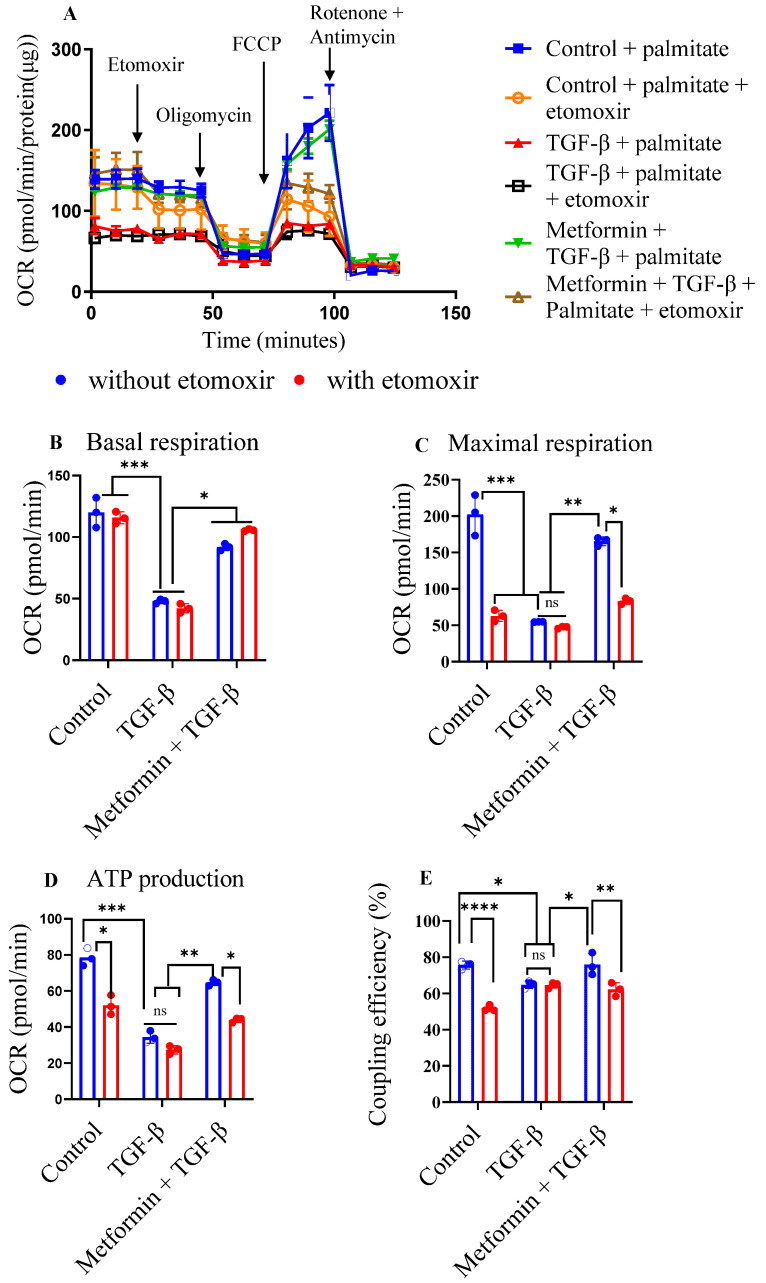
Metformin-mediated increase in mitochondrial oxygen consumption rate (OCR) is due to enhanced FAO in cardiac fibroblasts. (**A**) Adult mouse cardiac fibroblast cells were pretreated with or without metformin (2 mM) for 12 h followed by TGF-β (10 ng/mL) for 48 h and palmitate-BSA fatty acid oxidation substrate assay was performed to measure the mitochondrial OCR as described in methods. (**B**–**D**) Same as *A*, except that basal respiration (**B**), maximal respiration (**C**), ATP production (**D**) and coupling efficiency (**E**) were measured from the data obtained in *A*. GraphPad prism 10 was used to perform the statistics. The data shown in the graphs represent the mean ± SD of at least three different experiments. ns is non-significant, *, *p* ≤ 0.05; **, *p* ≤ 0.005; ***, *p* ≤ 0.0005; ****, *p* ≤ 0.0001 by two-way ANOVA followed by Tukey’s multiple-comparison test.

To further validate these observations, we measured the aforementioned parameters under the same treatment conditions using the palmitate-advanced assay following the exogenous addition of palmitic acid (PA), a free fatty acid known to stimulate FAO. Etomoxir significantly attenuated the basal respiration, maximal respiration, ATP production, and coupling efficiency in PA-treated control cells, indicating the dependence of mitochondrial OCR on CPT1-mediated FAO (Figure 4A–E). In contrast, PA failed to restore basal respiration, maximal respiration, ATP production, and coupling efficiency in TGF-β-treated cells compared to PA-treated control cells, thus suggesting impaired utilization of exogenous fatty acids under these conditions (Figure 4A–E). Similar trends were observed in the presence of PA and etomoxir in TGF-β-treated cells (Figure 4A–E). This is consistent with the diminished CPT1 activity by TGF-β, as presented in Figure 1A–C. Notably, metformin markedly preserved basal respiration, maximal respiration, ATP production, and coupling efficiency in TGF-β-treated cells supplemented with PA, while etomoxir significantly attenuated these parameters (Figure 4A–E). Collectively, these findings further support that metformin improves mitochondrial energetics by enhancing CPT1-dependent FAO in mouse cardiac fibroblasts.

### 3.3. Etomoxir Blunts Metformin-Mediated Attenuation of Cardiac Fibroblast Activation and Cellular Senescence

**Figure 5:**
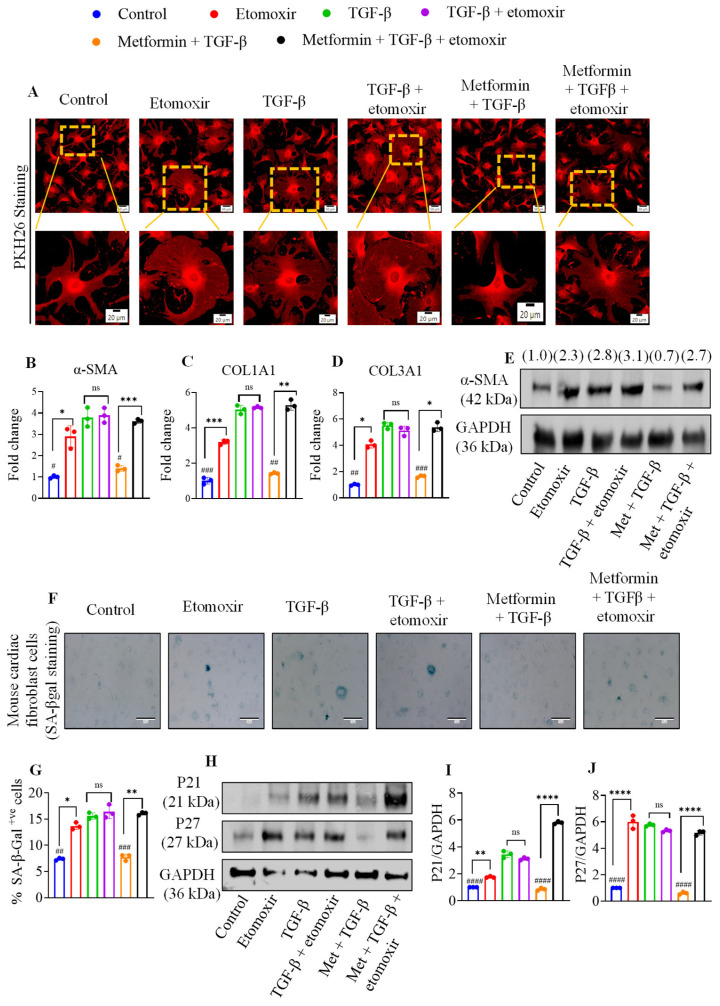
Metformin mitigates cardiac fibrosis markers and cellular senescence by promoting fatty acid β-oxidation. Adult mouse cardiac fibroblast cells were pretreated with metformin (2 mM) followed by treatment with TGF-β (10 ng/mL) in the presence or absence of etomoxir (40 µM) for 48 h. (**A**) Cells were labelled with PKH26 lipophilic dye and images were acquired using a laser scanning confocal microscope (scale bar = 50 µm). (**B**–**D**) Same as *A*, except that transcript levels of the indicated fibrosis markers were measured by RT-qPCR analysis. (**E**) Same as *A*, except that α-SMA protein levels were measured by immunoblotting. (**F**,**G**) Same as *A*, except that cellular senescence (**F**) was measured by SA-β-Gal staining (scale bar = 175 µm) and its quantification (**G**). (**H**) Same as *A*, except that senescence marker proteins like p21 and p27 were measured by immunoblot analysis. (**I**,**J**) Quantification of *H*. GraphPad Prism 10 was used for statistical analysis. Data are presented as mean ± SD from at least three independent experiments. ns is non-significant, *, *p* ≤ 0.05; **, *p* ≤ 0.005; ***, *p* ≤ 0.0005; ****, *p* ≤ 0.0001. #, *p* ≤ 0.05; ##, *p* ≤ 0.005; ###, *p* ≤ 0.0005; ####, *p* ≤ 0.0001 significantly different from TGF-β. Welch’s ANOVA followed by Dunnett’s T3 post hoc test for unequal variances and one-way ANOVA followed by Tukey’s multiple-comparison test for equal variances, depending on variance homogeneity.

We next investigated the impact of metformin’s ability to potentiate FAO in the regulation of TGF-β-induced cardiac fibroblast differentiation. For this, mouse cardiac fibroblasts were treated with metformin, etomoxir, or TGF-β, either individually or in combination. Morphological changes were initially assessed using PKH26 staining. TGF-β treatment caused cell enlargement, consistent with myofibroblast differentiation compared to a small, spindle-shaped morphology seen in control cells (Figure 5A). Notably, metformin markedly attenuated TGF-β-induced trans-differentiation, with cells retaining a morphology similar to untreated cells (Figure 5A). Interestingly, inhibition of FAO under these conditions by etomoxir diminished the protective effect of metformin against TGF-β-induced fibroblast activation (Figure 5A), indicating that metformin’s anti-fibrotic action is likely dependent on enhanced FAO. Accordingly, metformin treatment significantly reduced the transcript levels of fibrosis-associated markers like *α-SMA*, *COL1A1*, and *COL3A1* in TGF-β-treated cells, as well as α-SMA protein levels (Figure 5B–E). Furthermore, TGF-β-induced cardiac fibroblast differentiation coincided with the induction of cellular senescence as measured by senescence-associated β-Gal (SA-β-Gal) staining (Figure 5F,G). Metformin treatment significantly reduced TGF-β-induced senescence, as evidenced by a decreased number of SA-β-Gal-positive cells (Figure 5F,G). Intriguingly, etomoxir significantly abrogated metformin-mediated inhibition of premature senescence in cardiac fibroblast cells (Figure 5F,G). Similar trends were observed with senescence marker proteins like p21 and p27 by immunoblotting (Figure 5H–J). Thus, it appears that metformin, by potentiating the FAO pathway, alleviates cardiac fibroblast activation and the associated fibroblast senescence.

### 3.4. Metformin Administration Preserves Fatty Acid β-Oxidation Pathway in Aged Apoe^−/−^ Mice Heart: Effect on Age-Associated Cardiac Fibrosis Markers

**Figure 6:**
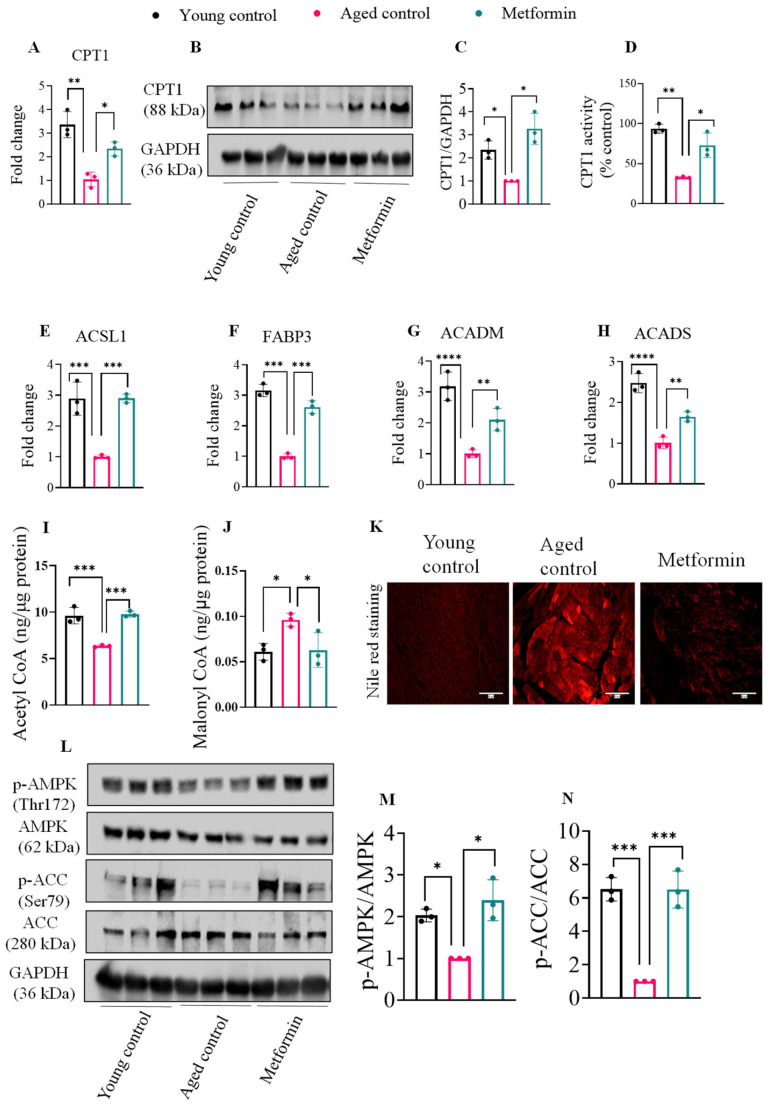
Metformin administration enhances CPT1 levels and fatty acid β-oxidation (FAO) pathway in the hearts of aged Apoe^−/−^ mice. (**A**–**D**) *CPT1* Transcript (**A**), protein (**B**,**C**), and enzyme activity (**D**) were measured in Apoe^−/−^ mice heart (n = 3 per group). (**E**–**H**) Transcript levels of *FABP3*, *ACSL1*, *ACADM*, and *ACADS* were measured in Apoe^−/−^ mice heart tissue by RT-qPCR analysis (n = 3 per group). (**I**,**J**) Acetyl-CoA (**I**) and Malonyl-CoA (**J**) levels were measured in Apoe^−/−^ mice heart tissue (n = 3 per group). (**K**) Lipid content was measured by Nile Red staining in Apoe^−/−^ mice heart sections and images were taken in a fluorescence microscope equipped with a rhodamine filter (n = 3 per group; scale bar = 85 µm). (**L**) Indicated protein levels were measured in Apoe^−/−^ mice heart tissue homogenates (n = 3 per group) by immunoblot analysis. (**M**,**N**) Quantification of *L*. GraphPad Prism 10 was used for statistical analysis. Data are presented as mean ± SD from at least three independent experiments. *, *p* ≤ 0.05; **, *p* ≤ 0.005; ***, *p* ≤ 0.0005; ****, *p* ≤ 0.0001. Welch’s ANOVA followed by Dunnett’s T3 post hoc test for unequal variances and one-way ANOVA followed by Tukey’s multiple-comparison test for equal variances, depending on variance homogeneity.

**Figure 7:**
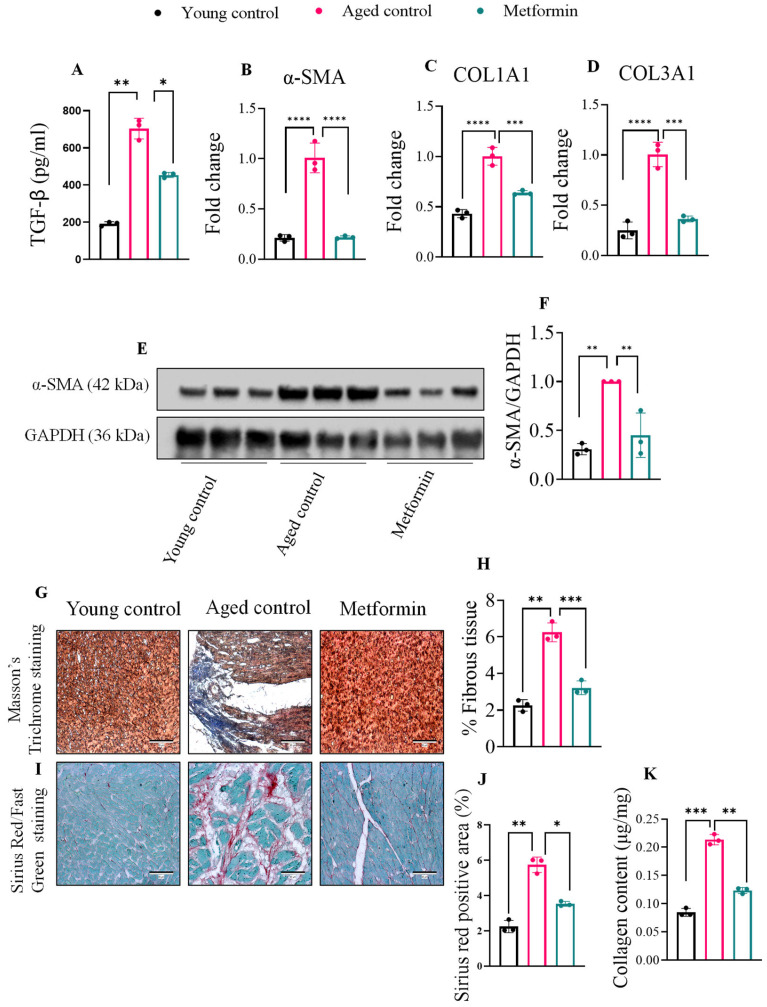
Metformin administration attenuates cardiac fibrosis markers in Apoe^−^/^−^ mice. (**A**) TGF-β levels were measured in Apoe^−/−^ mice heart tissue homogenates by ELISA (n = 3 per group). (**B**–**D**) Transcript levels of the indicated fibrosis markers were measured in Apoe^−/−^ mice heart tissue by RT-qPCR analysis (n = 3 per group). (**E**) α-SMA protein levels were measured in Apoe^−/−^ mice heart tissue homogenates by immunoblotting (n = 3 per group). (**F**) Quantification of *E*. (**G**) Masson’s trichome staining for collagen accumulation in the heart sections (n = 3 per group; scale bar = 85 µm). (**H**) Quantification of *G*. (**I**) Sirius Red/Fast Green staining for collagen accumulation in the heart sections (n = 3 per group; scale bar = 85 µm). (**J**) Quantification of *I*. (**K**) Total collagen content in heart tissues by hydroxyproline assay (n = 3 per group). GraphPad Prism 10 was used for statistical analysis. Data are presented as mean ± SD from at least three independent experiments. *, *p* ≤ 0.05; **, *p* ≤ 0.005; ***, *p* ≤ 0.0005; ****, *p* ≤ 0.0001. Welch’s ANOVA followed by Dunnett’s T3 post hoc test for unequal variances and one-way ANOVA followed by Tukey’s multiple-comparison test for equal variances, depending on variance homogeneity.

Cardiac fibrosis, in general, is associated with the progression of age. Aging and hypercholesterolemia are prominent and frequently overlapping contributors to cardiovascular dysfunction [18]. Studies in Apoe^−/−^ mice have demonstrated that chronic hypercholesterolemia coupled with aging promotes adverse metabolic remodeling, leading to heightened inflammation, cardiac hypertrophy, fibrosis, diminished cardiomyogenesis, and increased cardiomyocyte apoptosis [19,20]. To this end, to see if metformin administration improves age-induced alterations in FAO, and in turn, cardiac fibrosis markers, we performed an in vivo experiment in 12-month-old Apoe^−/−^ mice. Metformin (50 mg/kg body weight; a physiologically relevant dose) was administered daily via oral gavage for 12 weeks. In parallel, 6-month-old Apoe^−/−^ mice were included as a young control group alongside age-matched untreated control group. It was observed that CPT1 transcript and protein levels were significantly decreased in 12-month-old mice cardiac tissue compared to 6-month-old mice (Figure 6A–C). CPT1 enzyme activity followed a similar trend (Figure 6D), supporting an age-dependent decline in FAO capacity. Moreover, consistent with our cell culture results, metformin-treated mice cardiac tissue exhibited significantly elevated CPT1 levels compared to aged-matched Apoe^−/−^ control group (Figure 6A–D). In addition, metformin administration also enhanced the transcript levels of other FAO-related genes like *ACSL1*, *FABP3*, *ACADM*, and *ACADS* compared to aged-matched Apoe^−/−^ control group (Figure 6E–H), and are, in part, comparable to 6-month-old mice group. These results were further supported by elevated acetyl-CoA and diminished malonyl-CoA levels in metformin-administered mice group compared to age-matched control mice group (Figure 6I,J). Nile Red staining also revealed a noticeable reduction in lipid content in metformin-treated mice cardiac tissue sections compared to age-matched control mice cardiac tissue (Figure 6K). Similar to the results observed in cardiac fibroblast cells, metformin administration significantly increased AMPK activation and ACC phosphorylation (inhibitory phosphorylation) in cardiac tissue homogenates (Figure 6L–N), thus suggesting that metformin, by enhancing the AMPK–ACC axis, potentiates FAO in aged Apoe^−/−^ mice. To further determine if metformin influences the aging-associated cardiac profibrotic signaling, we measured TGF-β levels in heart tissue homogenates by ELISA. Metformin administration caused a significant reduction in aging-associated TGF-β levels (Figure 7A). These results were paralleled with a significant reduction in the profibrotic markers like α-SMA, COL1A1, and COL3A1 in metformin-administered mice heart tissues compared to age-matched control mice group (Figure 7B–F). The aforementioned profibrotic marker levels in metformin treated group are comparable to 6-month-old mice group (Figure 7B–F). Consistent with these findings, Masson’s trichrome and Sirius Red/Fast Green staining demonstrated greater collagen deposition in the hearts of aged-Apoe^−/−^ control mice compared with 6-month-old mice. Notably, metformin administration markedly attenuated collagen deposition (Figure 7G–J). These observations were further validated by hydroxyproline levels in the heart tissues (Figure 7K). Overall, these results indicate that metformin alleviates cardiac fibroblast activation in the heart tissue and improves age-associated cardiac fibrosis, in part, by preserving FAO.

## 4. Discussion

Cardiac fibrosis is a hallmark of cardiovascular aging and represents a major determinant of myocardial stiffness, ventricular dysfunction, and heart failure. Although accumulating evidence implicates metabolic dysfunction as a key driver of age-associated cardiac remodeling, the metabolic mechanisms governing cardiac fibroblast activation remain incompletely understood. In the present study, we demonstrate that metformin suppresses TGF-β-induced cardiac fibroblast activation and senescence by restoring CPT1-dependent fatty acid β-oxidation (FAO) through activation of the AMPK–ACC signaling axis. Furthermore, metformin preserved FAO and attenuated fibrosis-associated markers in aged Apoe^−/−^ mice, identifying FAO as a critical regulator of fibroblast fate and cardiac fibrotic remodeling during aging.

Fibroblast activation is increasingly recognized as a metabolically demanding process requiring extensive cellular reprogramming. Recent advances have highlighted that fibroblast activation is accompanied by dynamic metabolic adaptations, including changes in mitochondrial function, substrate utilization, and lipid metabolism, suggesting that metabolic state is not merely a consequence of fibrosis, but an active regulator of fibroblast phenotype [21,22]. Consistent with these reports, TGF-β-treated cardiac fibroblasts in our study exhibited reduced CPT1 expression and activity, diminished expression of FAO-related genes, and impaired mitochondrial respiration. Metformin effectively reversed these changes, restoring CPT1 activity, enhancing expression of *ACSL1*, *FABP3*, *ACADM*, and *ACADS*, and improving mitochondrial bioenergetics. Furthermore, in addition to enhancing FAO, metformin suppressed the TGF-β-induced expression of glycolytic markers, including *HIF-1α*, *GLUT1*, and *LDHA*, suggesting that metformin favors a metabolic profile associated with increased FAO and reduced glycolysis during cardiac fibroblast differentiation. These findings suggest that maintenance of FAO is essential for preserving fibroblast metabolic homeostasis and preventing pathological activation.

Several studies have demonstrated that metformin exerts anti-fibrotic effects in the heart, kidney, liver, and lung, primarily through activation of AMPK and inhibition of profibrotic pathways such as TGF-β/Smad signaling, NF-κB activation, and oxidative stress responses [23,24,25,26]. In experimental models of cardiac fibrosis, metformin attenuates myofibroblast differentiation and extracellular matrix deposition through AMPK-dependent suppression of TGF-β signaling [25]. In tune with this, in the current study, we observed that metformin suppresses TGF-β levels predominantly through activation of the AMPK signaling pathway in cardiac fibroblast cells. Additionally, in an independent lab finding, we observed that metformin attenuated TGF-β-induced cardiac fibroblast proliferation, suggesting that its anti-fibrotic actions extend beyond inhibition of myofibroblast differentiation. Studies in renal and pulmonary fibrosis have demonstrated that activation of AMPK by metformin limits fibroblast activation and tissue remodeling [23,26]. These findings are consistent with previous reports describing the cardiovascular beneficial effects of metformin through AMPK-dependent mechanisms, including attenuation of pathological remodeling and improvement of cardiovascular outcomes [27]. However, the downstream metabolic mechanisms responsible for these beneficial effects have remained largely unresolved. Our findings extend these observations by identifying FAO as a key metabolic effector downstream of AMPK. Specifically, metformin increased AMPK phosphorylation and inhibitory phosphorylation of ACC, resulting in reduced malonyl-CoA levels, enhanced CPT1 activity, and increased fatty acid utilization. Thus, beyond its established effects on profibrotic signaling pathways, metformin appears to reprogram fibroblast metabolism toward a more oxidative phenotype that is resistant to activation and senescence.

CPT1 is the rate-limiting enzyme responsible for mitochondrial transport of long-chain fatty acids, and plays a central role in maintaining cardiac metabolic flexibility. Aging-associated declines in CPT1 activity and FAO have been linked to mitochondrial dysfunction, lipid accumulation, and impaired cardiac energetics [28,29]. In agreement with these observations, aged Apoe^−/−^ mice exhibited reduced CPT1 expression and activity together with increased fibrotic remodeling. Notably, metformin restored CPT1 activity, reduced intracellular lipid accumulation, and increased acetyl-CoA levels both in vitro and in vivo. Importantly, pharmacological inhibition of CPT1 with etomoxir largely abolished the beneficial effects of metformin on mitochondrial respiration, ATP production, fibroblast activation, and senescence. These findings provide direct functional evidence that enhanced FAO is required for metformin-mediated cardioprotection.

Another important finding of the present study is the close relationship between FAO and cellular senescence. Senescent cardiac fibroblasts contribute to age-related fibrosis through the senescence-associated secretory phenotype, which promotes chronic inflammation and extracellular matrix deposition [30,31]. Accumulating evidence suggests that therapeutic targeting or elimination of senescent fibroblast populations can attenuate age-associated tissue fibrosis, supporting cellular senescence as an attractive therapeutic target in chronic fibrotic disorders [32,33]. In the present study, metformin significantly attenuated TGF-β-induced SA-β-Gal^+ve^ fibroblast cells and reduced p21 and p27 levels, whereas inhibition of CPT1 reversed these effects. These observations suggest that impaired FAO may act as a metabolic trigger linking mitochondrial dysfunction to fibroblast senescence and fibrotic remodeling. Interestingly, these findings are consistent with our previous studies demonstrating that metformin delays endothelial senescence through AMPK-dependent mechanisms involving mitochondrial biogenesis, H3K79 trimethylation, and enhanced FAO [11,13]. Together, these studies support the concept that restoration of mitochondrial metabolism is a common mechanism underlying the geroprotective actions of metformin across multiple cardiovascular cell types.

The physiological relevance of these observations was further validated in aged Apoe^−/−^ mice, a model characterized by hypercholesterolemia, metabolic dysfunction, and accelerated cardiovascular aging [20,34]. Metformin restored FAO-related gene expression, enhanced CPT1 activity, activated AMPK signaling, and significantly reduced cardiac fibrosis markers. These findings indicate that preservation of FAO contributes substantially to the anti-fibrotic effects of metformin during aging-associated cardiac remodeling. While these results are interesting, one of the limitations of the present study is the use of aged Apoe^−/−^ mice, which exhibit dyslipidemia and enhanced atherosclerotic susceptibility and therefore do not completely model physiological aging. Moreover, few studies have employed additional pro-fibrotic stimuli, such as high-fat diet feeding or angiotensin II infusion, to induce more pronounced cardiac fibrosis in Apoe^−/−^ mice [35,36]. Although this model is relevant to aging-associated cardiovascular disease [20], the contribution of ApoE deficiency and associated metabolic and vascular abnormalities to cardiac fibrosis cannot be fully excluded. Accordingly, the present findings should be interpreted in the context of aging-associated cardiac fibrosis under atherosclerosis-prone conditions. Future studies using naturally aged wild-type mice will be important to determine whether metformin exerts comparable anti-fibrotic effects during normal aging. Another limitation of this study is the relatively small number of biological samples used for individual in vivo analyses. Nevertheless, the consistency of findings across complementary histological, molecular, and biochemical approaches supports the overall conclusions. Additionally, in this study, we have not assessed the in vivo cardiac functional parameters. Although metformin reduced myocardial collagen accumulation and the expression of pro-fibrotic markers, these findings do not directly establish an improvement in cardiac performance. Future studies incorporating comprehensive cardiac functional assessment will be necessary to determine whether the structural anti-fibrotic effects of metformin translate into improved systolic and/or diastolic function in aged Apoe^−/−^ mice.

## 5. Conclusions

The present study identifies CPT1-dependent FAO as a previously underappreciated metabolic regulator of cardiac fibroblast activation and senescence. Our findings establish the AMPK–ACC–CPT1 pathway as a critical mechanistic link between metformin treatment and suppression of cardiac fibrosis. By restoring mitochondrial fatty acid metabolism and cellular bioenergetics, metformin limits fibroblast activation and age-associated fibrotic remodeling. These results not only provide new mechanistic insight into the cardioprotective actions of metformin, but also highlight targeting FAO as a promising therapeutic strategy for combating age-related cardiac fibrosis.

## Figures and Tables

**Table 1:** List of Mouse Primer Sets Used.

Gene	Forward Primer	Reverse Primer
CPT1	AAGTTCTGCCTGACTACGAGG	ATGTTTTGGTGCTTTTCGGAGG
FABP3	CATCATCGAGAAGAACGGGGAT	TTCTGCACATGGATGAGTTTGC
ACSL1	CATCGTCAGAAACAACAGCCTG	GGTCTGTCCGTAGCCTTCATAG
ACADM	CGGAACACTTACTATGGGTCGA	CTGATAGATCTTGGCGTCCCTC
ACADS	CTCCTCCACAGCTAACCTCATC	GGCATACTTCACAGCACAATCC
α-SMA	CTATTCCTTCGTGACCACAGCT	CCGCAGACTCCATACCGATAAA
COL1A1	CTCAAGATGTGCCACTCTGACT	ACCTGTCTCCATGTTGCAGTAG
COL3A1	ACGTAAGCACTGGTGGACAG	GGAGGGCCATAGCTGAACTG
18S	AGTTATGGTTCCTTTGGTCGCT	TTATCTAGAGTCACCAAGCCGC

## Data Availability

The data that support the findings of this study are available from the corresponding author upon reasonable request.

## Abbreviations

The following abbreviations are used in this manuscript:

FAO	Fatty acid β-oxidation
TGF-β	Transforming growth factor-β
CPT1	Carnitine palmitoyltransferase-1
OCR	Mitochondrial oxygen consumption rate
AMPK	AMP-activated protein kinase
ACC	Acetyl-CoA carboxylase
FABP3	Fatty Acid-Binding Protein 3
ACSL1	Acyl-CoA Synthetase Long Chain Family Member 1
ACADM	Medium-Chain Acyl-CoA Dehydrogenase
ACADS	Short-chain acyl-CoA dehydrogenase
α-SMA	Alpha-Smooth Muscle Actin
COL1A1	Collagen Type I Alpha 1 Chain
COL3A1	Collagen Type III Alpha 1 Chain
